# Chemokine Receptor N-Terminus Charge Dictates Reliance on Post-Translational Modifications for Effective Ligand Capture and Following Boosting by Defense Peptides

**DOI:** 10.3390/ijms251910854

**Published:** 2024-10-09

**Authors:** Ting Xu, Anne Sophie Schou, Jarkko J. Lackman, Marina Barrio-Calvo, Lisa Verhallen, Christoffer Knak Goth, Benjamin Anderschou Holbech Jensen, Christopher T. Veldkamp, Brian F. Volkman, Francis C. Peterson, Gertrud Malene Hjortø

**Affiliations:** 1 Department of Biomedical Sciences, University of Copenhagen, 2200 Copenhagen, Denmark; serenezka@gmail.com (T.X.); annesschou@gmail.com (A.S.S.); barriocalvo.marina@gmail.com (M.B.-C.); lisa.verhallen@kuleuven.be (L.V.); christoffer.goth@hotmail.com (C.K.G.); benjamin.jensen@sund.ku.dk (B.A.H.J.); 2 Copenhagen Center for Glycomics, University of Copenhagen, 2200 Copenhagen, Denmark; jajl@sund.ku.dk; 3 Evaxion Biotech, 2970 Hørsholm, Denmark; 4 Department of Microbiology, Immunology and Transplantation, KU Leuven, 3000 Leuven, Belgium; 5 Glx Analytix APS, 2400 Copenhagen, Denmark; 6 Department of Chemistry, University of Wisconsin-Whitewater, Whitewater, WI 53190, USA; veldkamc@uww.edu; 7 Department of Biochemistry, Medical College of Wisconsin, Milwaukee, WI 53226, USA; bvolkman@mcw.edu (B.F.V.); fpeterso@mcw.edu (F.C.P.)

**Keywords:** receptor N-terminus, CCR1, CCR5, electrostatic interaction, post-translational modification, glycosaminoglycan, signaling, chemokine, O-glycosylation, tyrosine sulfation

## Abstract

The chemokine receptors CCR1 and CCR5 display overlapping expression patterns and ligand dependency. Here we find that ligand activation of CCR5, not CCR1, is dependent on N-terminal receptor O-glycosylation. Release from O-glycosylation dependency is obtained by increasing CCR5 N-terminus acidity to the level of CCR1. Ligand activation of CCR5, not CCR1, drastically improves in the absence of glycosaminoglycans (GAGs). Ligand activity at both CCR1 and CCR5 is boosted by positively charged/basic peptides shown to interact with acidic chemokine receptor N-termini. We propose that receptors with an inherent low N-terminus acidity rely on post-translational modifications (PTMs) to efficiently compete with acidic entities in the local environment for ligand capture. Although crucial for initial ligand binding, strong electrostatic interactions between the ligand and the receptor N-terminus may counteract following insertion of the ligand into the receptor binding pocket and activation, a process that seems to be aided in the presence of basic peptides. Basic peptides bind to the naked CCR1 N-terminus, not the CCR5 N-terminus, explaining the loss of boosting of ligand-induced signaling via CCR5 in cells incapable of glycosylation.

## 1. Introduction

The human chemokine system encompasses 23 receptors and ~50 chemokines [1]. It is by nature a selectively promiscuous system, with most receptors being activated by more than one ligand and by a high degree of ligand sharing across a subset of receptors [2]. The chemokine receptors CCR1 and CCR5 display overlapping expression patterns and ligand dependency. Both receptors are activated by CCL3, CCL4, CCL5, CCL8, CCL13, CCL14, and CCL16 [3,4,5,6]. CCR5 is not activated by other chemokines, whereas CCR1 is additionally activated by CCL7, CCL15, and CCL23 [4,7,8]. In the current study we find that post-translational modifications (PTMs) of receptor N-termini differentially regulate ligand activity at CCR5 versus CCR1. Most chemokines are highly basic, permitting them to be sequestered by sulfated glycosaminoglycans (GAGs) on the cell surface and extracellular matrix (ECM), creating local chemokine stores [9,10,11]. According to the chemokine cooperativity model, abundant chemokines mobilize less abundant chemokines from GAG-bound stores, increasing their availability for receptor interactions and concomitant immune cell stimulation [9]. In the current study we show that CCR1 with a high content of acidic amino acids (aa) in the receptor N-terminus relies less on PTMs for efficient receptor activation compared to CCR5 that harbors an N-terminus with low inherent negative charge. Thus, we propose that chemokine receptors with acidic N-termini compete effectively with GAGs for chemokine sequestering, whereas receptors with less acidic N-termini rely heavily on PTMs for adding to the overall negative charge of the receptor N-terminus and thus for effective ligand capture and immune cell stimulation. According to the two-site–two-step model, ligand-induced GPCR activation is governed by a sequential binding and activation mechanism involving initial ligand capture followed by proper docking of the ligand into the receptor binding pocket causing changes in receptor conformation allowing for coupling to intracellular signaling modalities [12]. Following this model, the chemokine core domain referred to as chemokine site 1 (CS1) interacts with the N-terminus and extracellular loops (ECLs) of the chemokine receptor known as chemokine receptor site 1 (CRS1). After this initial binding step, the chemokine N-terminus referred to as chemokine site 2 (CS2) is inserted into the receptor binding pocket in the transmembrane space (CSR2) [12]. Our results are in line with the model previously put forward by Sanchez et al. proposing that initial chemokine binding to a receptor involves low-affinity CS1:CRS1 interactions that are non-specific and reversible, followed by a specific rate-limiting, high-affinity interaction between CS2 and CRS2, leading to docking of the chemokine N-terminus into the receptor binding pocket resulting in receptor conformational changes and activation [8].

We further propose that basic peptides, like the naturally occurring C-terminal peptide of CCL21 (C21TP^81-111^), aid the transition from the initial CS1:CRS1 interaction to the insertion of the chemokine N-terminus (CS2) deep into the binding pocket of the chemokine receptor (CRS2), through shielding of the acidic receptor N-terminus. Basic peptides play important roles in immune activation [13], and we observe similar boosting effects in the presence of human beta defensin 2 (hBD2), that is present in micromolar concentrations in inflamed tissues [14]. C21TP^81-111^, as well as shorter versions thereof, are generated by proteases secreted by dendritic cells (DCs) [15] and by proteases like plasmin presented on the cell membrane of, e.g., T cells [16,17]. This generates a version of CCL21 that is much more potent than the full-length chemokine [18,19], but at the same time, the basic tail peptide fragments, shown by us to potently boost the action of full-length CCL21 [19,20], could play a role in controlling the activity of CCL21 and CCL19 in the immunological synapse affecting the on and off rate of the CS1:CRS1 interaction. Also, as the C-terminus of CCL21 is known to interact with, e.g., polysialic acid PTMs present in both the receptor for CCL21 (CCR7) [21] and in the co-receptor NRP2 [22,23] expressed by DCs, other regulatory mechanisms could be affected by basic peptides generated from chemokines through proteolytic activity or released by neutrophils at the site of infection (e.g., hBD2).

## 2. Results

### 2.1. Basic Peptide Boosting of Ligand-Induced Signaling at CCR1 and CCR5

To test if basic peptides boost signaling of basic chemokine ligands targeting CCR5 and CCR1, we investigated Gα_i_ signaling induced by two shared ligands for CCR5 and CCR1 (CCL3 and CCL5) and one cognate ligand of each receptor (CCL8 for CCR5 and CCL7 for CCR1), in the absence or presence of a basic peptide. The basic peptide used here corresponds to the last 31 aa (residues 81-111) of the chemokine CCL21, hereafter referred to as C21TP^81-111^. Basic chemokines like CCL5 contain high amounts of the amino acids arginine (R) and lysine (K) that are positively charged at physiological pH. CCL3 is rather acidic, as it contains more glutamic acid (E) and aspartic acid (D) amino acids that are negatively charged at physiological pH. Thus, CCL5 carries five E/D in total and ten R/K in total, making it overall basic (positively charged). CCL3 carries eight E/D in total and six R/K in total, making it overall acidic (negative in charge).

For quantification of signaling initiated immediate downstream of receptor activation, we used the cAMP binding CAMYEL sensor to perform bioluminescence resonance energy transfer (BRET)-based assays. Shortly, in this assay increased receptor activation leads to an increase in BRET ratio. For detailed information see the Materials and Methods section.

In signaling assays testing the boosting ability of basic peptides, transfected cells were aliquoted into 96-well plates in the presence of 10 µM peptide or PBS vehicle. After R-Luc substrate addition, chemokines were added to reach final concentrations ranging from 0–100 nM (0, 0.1, 1, 10, and 100 nM) and forskolin was applied before reading emission at 475 and 525 nm.

**Figure 1:**
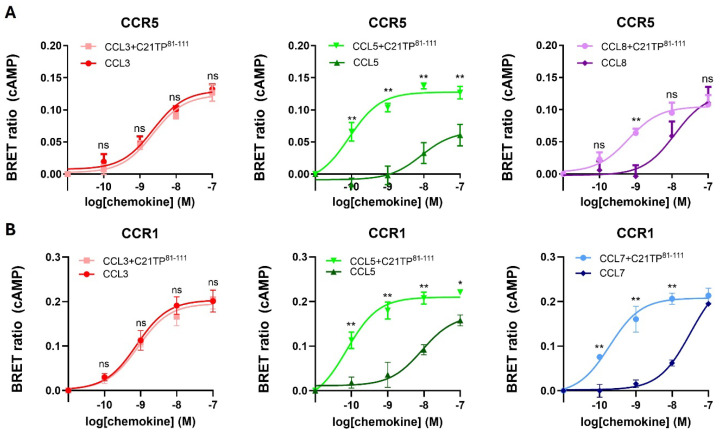
Basic peptides boost signaling induced by basic chemokines, with no effect on signaling induced by acidic chemokines, at both CCR1 and CCR5. Ligand-induced Gα_i_ signaling via CCR5 and CCR1 in the absence or presence of C21TP^81-111^ measured in CHO-K1 cells transiently transfected to express the chemokine receptors and the cAMP sensor CAMYEL (EPAC). (**A**) C21TP^81-111^ boosts the potency of CCL5 and CCL8 in Gα_i_ signaling but has no effect on CCL3-induced signaling via CCR5. (**B**) Signaling induced by CCL5 and CCL7, but not CCL3, via CCR1 is boosted in the presence of C21TP^81-111^. Statistical significances were determined by two-way ANOVA with Sidak’s multiple comparisons test (number of independent experiments, n = 3). ** *p* ≤ 0.01, * *p* ≤ 0.05, ns = non-significant.

As can be observed in Figure 1, ligand-induced signaling at CCR5 was boosted by C21TP^81-111^ in the case of CCL5 and CCL8 (Figure 1A) and, similarly, ligand-induced signaling at CCR1 was boosted by C21TP^81-111^ in the case of CCL5 and CCL7 (Figure 1B). These data indicate that the more basic the chemokine the more boosting by the basic peptide, whereas signaling induced by the acidic chemokine, CCL3, was not affected by C21TP^81-111^ (Figure 1A,B).

**Figure 2:**
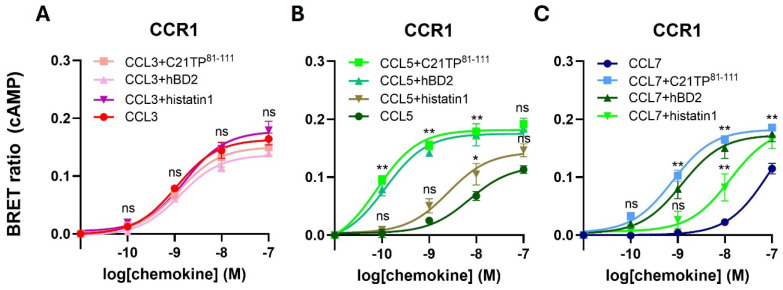
Apart from the chemokine-derived peptide C21TP^81-111^, basic peptides in the form of host defense peptides also boost signaling of basic chemokines. Ligand-induced Gα_i_ signaling via CCR1 in the absence or presence of C21TP^81-111^, hBD2, and histatin1 measured in CHO-K1 cells transfected as in Figure 1. (**A**) None of the basic peptides affected CCL3-induced signaling via CCR1. Statistical significances were determined by two-way ANOVA with Sidak’s multiple comparisons test (n = 3). (**B**,**C**) hBD2 boosts the potency of CCL5 and CCL7 in Gα_i_ signaling via CCR1 to the same extent as C21TP^81-111^. Histatin1 also boosts the potency of CCL5 and CCL7, but only to a minor degree. ** *p* ≤ 0.01, * *p* ≤ 0.05, ns = non-significant.

To test if other basic peptides hold boosting potential towards basic chemokines, human anti-microbial peptides (AMPs), also known as host defense peptides (HDPs), human beta defensin 2 (hBD2), and histatin1 was tested in signaling induced by CCL3, CCL5, and CCL7 via CCR1. hBD2 were as efficient in boosting signaling via CCR1 by CCL5 and CCL7 as C21TP^81-111^ (Figure 2B,C). Histatin1, that is less basic than hBD2, also boosted the potency of both these chemokines, although to a lesser extent. Interestingly, CCL3 signaling was not affected by any of the basic peptides tested (Figure 2A).

### 2.2. GAG Interference with Ligand-Induced Signaling at CCR5, Not at CCR1

**Figure 3:**
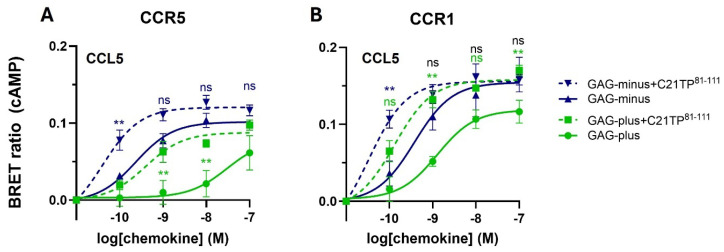
CCL5 signaling through CCR5 and to a lesser extent CCR1 is improved in the absence of GAGs. Ligand-induced Gα_i_ signaling via CCR5 and CCR1 in the absence or presence of C21TP^81-111^ in CHO GAG-minus and CHO GAG-plus cells transfected as in Figure 1. (**A**) Both the potency and the efficacy of CCL5 are significantly increased in GAG-minus cells compared to GAG-plus cells in the absence of C21TP^81-111^ boosting. Thus, although boosting persists in the GAG-deficient cells, it is limited how much boosting can occur when signaling in the absence of basic peptides is already very potent. (**B**) In contrast to CCL5 signaling via CCR5, signaling induced by the same ligand via CCR1 is potent in both GAG-deficient and WT CHO cells, although signaling in CHO deficient cells still exceeds that observed in WT CHO cells. Again, since signaling is potent in both GAG-plus and GAG-minus cells, boosting exerted by C21TP^81-111^although significant, is limited. Statistical significances were determined by two-way ANOVA with Sidak’s multiple comparisons test (n = 3). ** *p* ≤ 0.01, ns = non-significant.

As the C21TP^81-111^ boosting effect has previously been shown to be independent of cell GAGs, signaling of CCL5 via CCR5 and CCR1 was tested in CHO cells completely deficient in GAG synthesis due to a lack of endogenous B4GalT7 activity (CHO-pgsB618, referred to as GAG-minus). As observed in Figure 3A, CCL5 signaling via CCR5 is boosted by C21TP^81-111^ in both GAG-plus (WT) and GAG-minus cells. Interestingly, CCL5 on its own induces signaling that is significantly more potent and efficacious in cells that do not have GAGs compared to cells with GAGs (Figure 3A). Signaling induced by CCL5 via CCR1 in general is more potent and efficacious than signaling induced by the same ligand via CCR5. Again, boosting occurs in both cell lines. More importantly, signaling seems to not be reliant on cell surface GAG expression, and CCL5 signaling via CCR1 is very potent in both GAG-plus and GAG-minus cells (Figure 1B).

### 2.3. PTMS Are Needed for Ligand-Induced Signaling at CCR5, Not at CCR1

Chemokine receptor N-terminal acidity is modulated by PTMs in the form of O- and N-glycosylation as well as tyrosine sulfation [24]. Tyrosine sulfation has previously been shown to be essential for CCL3, CCL4, and CCL5 binding to and activation of CCR5 [6,25]. In the current study we confirm that tyrosine sulfations are important for CCL5 signaling via CCR5, using mutated CCR5 constructs with alanine substitutions at tyrosine 10 (Y10A) and Y14/15 (Y1415AA), respectively. CCL5 signaling via Y10A-CCR5 is significantly reduced compared to CCL5 signaling via WT-CCR5 (Supplementary Materials Figure S1A) and completely abolished in Y14Y15AA-CCR5 and the triple mutant Y10Y14Y15AAA-CCR5 (Supplementary Materials Figure S1B,C). Boosting by C21TP^81-111^ was maintained in both Y10A and Y1415AA receptor variants, but not in the Y10Y14Y15AAA receptor variant. In a similar manner we tested the importance of O-glycosylations for ligand-induced signaling by alanine substitutions at S6/S7 (S6S7AA), T16/S17 (T16S17AA), and S6S7T16S17 (S6S7T16S17AAAA). Again, CCL5 signaling via S6S7AA-CCR5 was significantly reduced (Supplementary Materials Figure S2A). Interestingly, but in line with the previous study focusing on CCL3 and CCL4 [25], CCL5 signaling via T16S17AA-CCR5, was improved compared to WT-CCR5 (Supplementary Materials Figure S2B) whereas CCL5 signaling via S6S7T16S17AAAA-CCR5 was completely abolished (Supplementary Materials Figure S2C). Boosting by C21TP^81-111^ was maintained in S6S7AA and T16S17AA, but not in S6S7T16S17AAAA (Supplementary Materials Figure S2A–C).

**Figure 4:**
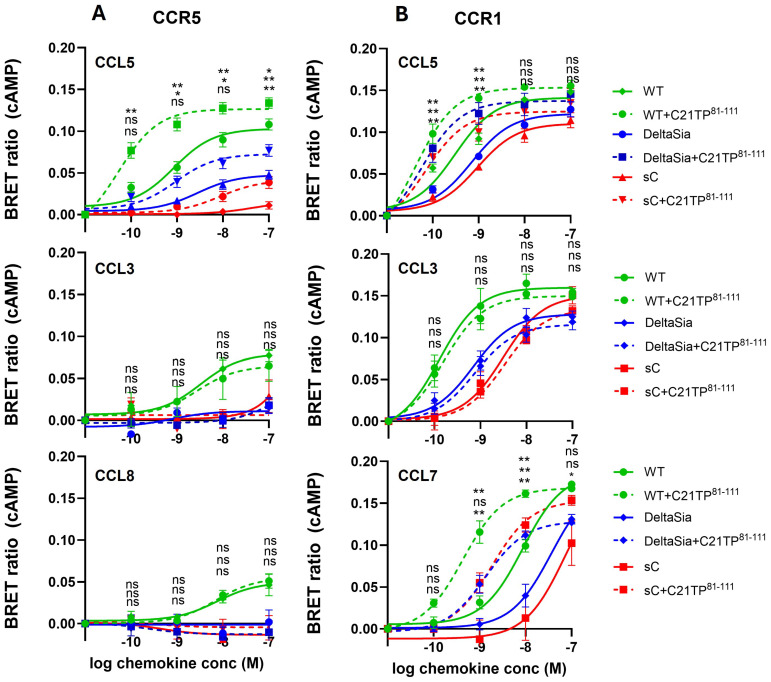
Chemokine signaling via CCR5, not CCR1, is highly dependent on receptor O-glycosylation. Ligand-induced Gα_i_ signaling via CCR5 and CCR1 in the absence or presence of C21TP^81-111^ in HEK293 WT, DeltaSia, and sC cells transfected as in Figure 1. (**A**) Compared to CCL5 signaling in WT cells, both potency and efficacy of CCL5 significantly drop in DeltaSia cells and even more so in sC, while C21TP^81-111^ boosting seems to persist in all cell lines. CCL3- and CCL8-induced signaling is drastically reduced in DeltaSia and sC cells and boosting of CCL8 disappears. (**B**) In contrast, CCL5, CCL3, and CCL7 signaling via CCR1 persists in DeltaSia and sC to almost the same high level as observed in WT cells. Boosting of CCL5 and CCL7 signaling also persists. Signaling by CCL3, as expected, is not boosted in the presence of C21TP^81-111^. Statistical significances were determined by two-way ANOVA with Sidak’s multiple comparisons test (n = 3). ** *p* ≤ 0.01, * *p* ≤ 0.05, ns = non-significant.

In general, ligand-induced signaling via CCR5 seems to be heavily regulated at the post-translational level, in the form of both tyrosine sulfation and O-glycosylation [6,25,26]. To further study the role of O-glycosylation we tested signaling induced at CCR1 and CCR5 side by side in WT, DeltaSia, and sC HEK293 cells. As can be seen in Figure 4, CCL5 signaling via CCR5 in HEK293 cells (Figure 4, upper panel) is more potent than in CHO cells (Figure 1A, middle panel). Interestingly, CCL5 signaling via CCR5 in HEK293 cells is significantly impaired in DeltaSia cells and almost abolished in sC (Figure 4, upper panel), indicating that terminal sialic acids are important for ligand–receptor interaction. Boosting by C21TP^81-111^ was observed in all three cell lines. Signaling by CCL3 and CCL8 via CCR5 is only maintained in WT cells and boosting by C21TP^81-111^ is not only absent for CCL3 but also for CCL8 (Figure 4A, middle and lower panels). As can be seen in Figure 4B, upper panel, CCL5 signaling via CCR1 seems to be independent of O-glycosylation and CCL5 signals potently through CCR1 in WT, DeltaSia, and sC and C21TP^81-111^ boosting is preserved across all three cell lines.

**Figure 5:**
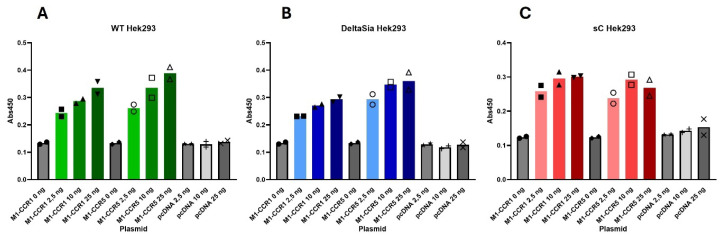
Receptors are expressed at the same level on the surface of WT, DeltaSia, and sC HEK293. Cell surface expression levels of CCR1 and CCR5 determined using M1-tagged receptors (**A**–**C**). M1-CCR1 and M1-CCR5 are expressed at the cell surface at similar levels to each other within all three cell lines, HEK293 WT (green columns, **A**), DeltaSia (blue columns, **B**), and sC (red columns, **C**), transfected as in Figure 1, but without CAMYEL. The data are from one experiment with each column representing the mean of two duplicate datasets. No *p* values are calculated. The experiment represents one out of two independent experiments.

To confirm that the receptors are expressed equally well at the cell surface in all three cell lines, WT, DeltaSia, and sC cells were transfected with increasing amounts of plasmid DNA encoding the CCR1 and the CCR5 receptor in an M1-tagged version to be able to follow surface expression upon staining with antibodies towards the M1 tag. We also transfected the cells with the empty pcDNA3.1 backbone plasmid. As can be observed in Figure 5, both receptors are expressed in a dose-dependent manner to equal levels in all three cell lines.

### 2.4. PTMs Are Needed for Peptide Interaction with CCR5, Not at CCR1 N-Terminus

**Figure 6:**
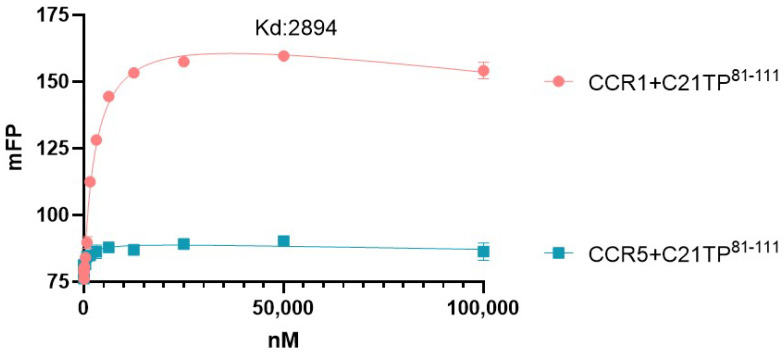
C21TP^81-111^ binds in a dose-dependent manner to the naked CCR1, not the naked CCR5 N-terminus. Fluorescence polarization of the CCR1 N-terminal peptide increased in a dose-dependent manner upon addition of the C21TP^81-111^, yielding a hyperbolic binding curve and a dissociation constant (*K*_d_) of ~2894 nM. No binding of C21TP^81-111^ to the CCR5 N-terminal peptide was observed. Datapoints with SEM (n = 3).

To investigate if the loss of boosting of ligand-induced signaling at CCR5 in DeltaSia and sC cells could be explained by loss of peptide affinity for the CCR5 N-terminus in the absence of functional glycosylation we measured the binding of C21TP^81-111^ to peptides corresponding to the naked CCR1 and CCR5 N-termini N-terminally tagged with fluorescein (FAM). Using fluorescence polarization assay, we found that the fluorescence polarization of the CCR1 N-terminal peptide increased in a dose-dependent manner upon addition of the C21TP^81-111^, yielding a hyperbolic binding curve and a dissociation constant (*K*_d_) of ~2894 nM (Figure 6). In contrast no such binding is observed for the naked CCR5 N-terminus (Figure 6). Like C21TP^81-111^, hBD2 and histatin1 were found to bind in a dose-dependent manner to the CCR1 N-terminus (hBD2 with a higher affinity than histatin1), whereas neither of the HDPs bound to the naked CCR5 N-terminus.

### 2.5. Inherent Charge of Receptor N-Terminus Dictates Dependence on PTMs for Ligand-Induced Signaling

**Figure 7:**
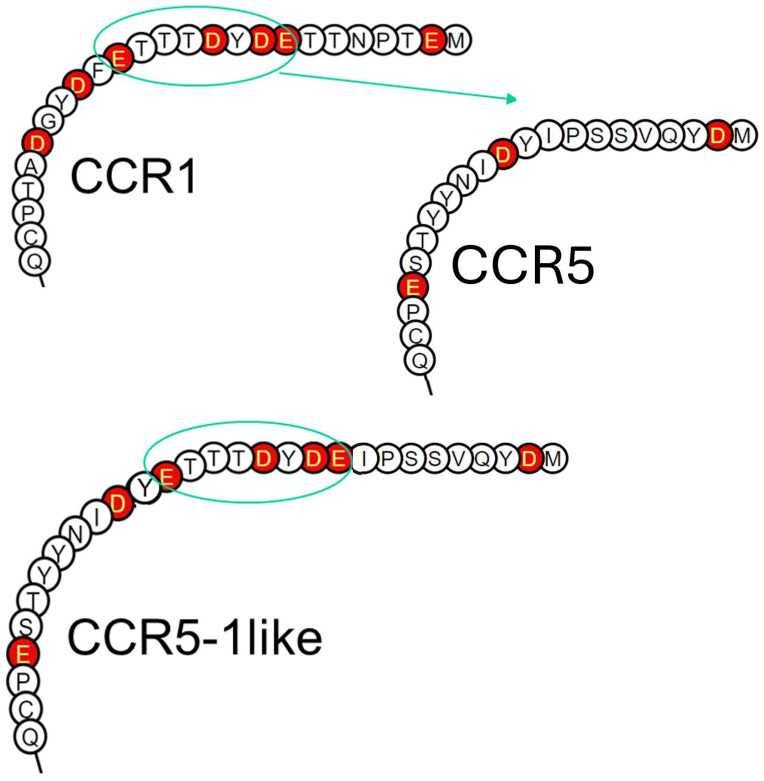
Receptor N-termini representing CCR1, CCR5, and the chimeric CCR5-1like. The CCR1 N-terminus contains a lot of acidic amino acids (outlined in red) in the form of aspartic acid (D) and glutamic acid (E), whereas CCR5 only contains a few. The chimeric receptor CCR5-1like that contains part of the CCR1 receptor N-terminus (ETTTDYDE) resembles CCR1 with regard to acidic amino acid content and spacing, but they are not the same.

To finally determine if the lack of acidic amino acids in the CCR5 N-terminus can explain the dependency of this receptor on O-glycosylation for effective ligand interaction, we constructed a CCR5 receptor chimera, where the most acidic N-terminal region of CCR1 was inserted into the CCR5 N-terminus, creating the chimeric receptor CCR5-1like (Figure 7).

**Figure 8:**
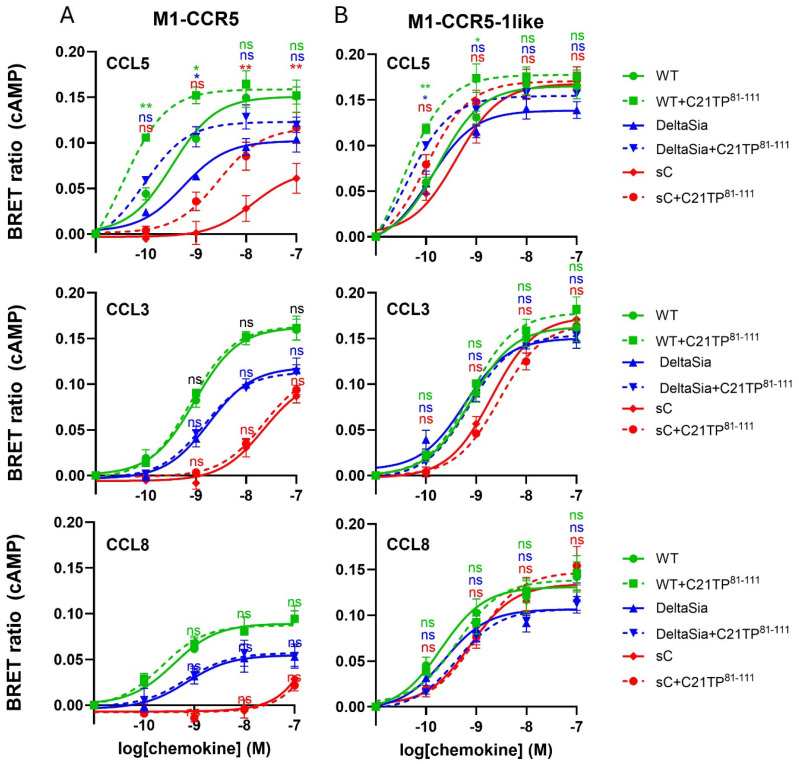
Chemokine signaling via CCR5-1like is independent of receptor O-glycosylation status. Ligand-induced Gα_i_ signaling via M1-CCR5 and M1-CCR5-1like in the absence or presence of C21TP^81-111^ in HEK293 WT, DeltaSia, and sC cells transfected as in Figure 1. (**A**) As was observed with the untagged CCR5 receptor, compared to CCL5 signaling in WT cells, both potency and efficacy of CCL5 significantly drop in DeltaSia cells and even more so in sC, while C21TP^81-111^ boosting seems to persist in all cell lines. CCL3- and CCL8-induced signaling via M1-CCR5 is drastically reduced in DeltaSia and sC cells and boosting of CCL3 and CCL8 is absent. (**B**) In contrast, CCL5, CCL3, and CCL8 signaling via M1-CCR5-1like persists in DeltaSia and sC to almost the same high level as observed in WT cells. C21TP^81-111^ boosting of CCL5 signaling persists in all three cell lines but is absent for CCL3 and CCL8. Statistical significances were determined by two-way ANOVA with Sidak’s multiple comparisons test (n = 3). ** *p* ≤ 0.01, * *p* ≤ 0.05, ns = non-significant.

Compared to CCL5 signaling via untagged CCR5 (Figure 4A, upper panel), CCL5 signaling via M1-CCR5 seems to be more potent (Figure 8A, upper panel), probably due to the M1 tag itself, as it adds additional negative charge to the receptor in the form of five aspartic acid residues and a putative tyrosine sulfation site (DYKDDDDK) positioned at the extreme N-terminus. Still, CCL5 signaling via M1-CCR5 was drastically reduced in DeltaSia cells and even more so in sC cells. CCL5-induced signaling via M1-CCR5 was boosted by C21TP^81-111^ in all three cell lines. As for CCL5, CCL3 and CCL8 signaling via M1-CCR5 (Figure 8A, middle and lower panels) seems to be more potent than via untagged CCR5 (Figure 4A, middle and lower panels). As observed with the untagged CCR5, signaling via M1-CCR5 induced by both CCL3 and CCL8 decreases going from WT to DeltaSia to sC, and signaling was not boosted by C21TP^81-111^. Importantly, for all three ligands, the signaling became much more potent and efficacious via the CCR5-1like receptor that had part of the CCR1 receptor inserted into the N-terminus, including the DYDE sequence, similar to DYDY, a sequence that is known to be extremely important for efficient ligand binding in CCR2 [27]. Thus CCL5, CCL3, and CCL8 signaling resembles the signaling induced via CCR1, with all ligands being equally potent in all three cell lines, WT, DeltaSia, and sC (Figure 8B). In fact, signaling was so potent that there was almost no room for boosting CCL5, and significant C21TP^81-111^ boosting was still observed in both WT and DeltaSia cells for CCL5 (Figure 8B, upper panel).

### 2.6. Receptor N-Terminus Serves to Capture Ligands, Not to Dictate Fruitful Ligand Docking and Receptor Activation

**Figure 9:**
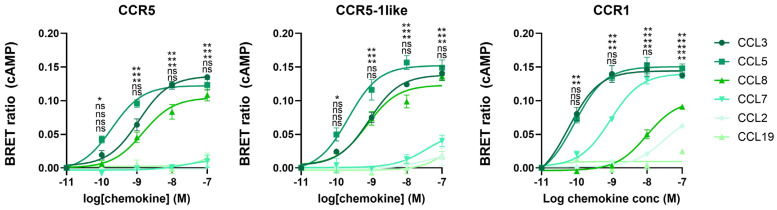
CCR5-1like retains specificity towards CCR5 cognate ligands. Ligand-induced Gα_i_ signaling via M1-CCR5, M1-CCR5-1like, and M1-CCR1 in HEK293 WT cells transfected as in Figure 1. As can be seen from the left and middle panels, CCR5 and CCR5-1like maintain the same signaling pattern, responding to CCR5 cognate ligands CCL3, CCL5, and CCL8, but not to CCL2, CCL7, and CCL19, as expected. In contrast CCR1, as expected, responds to a broader range of chemokines, including its cognate ligands CCL3, CCL5, and CCL7, but also CCL8 and to a minor degree CCL2. Statistical significances were determined by two-way ANOVA with Sidak’s multiple comparisons test (n = 3). ** *p* ≤ 0.01, * *p* ≤ 0.05, ns = non-significant.

To test if the chimeric receptor CCR5-1like has maintained CCR5 ligand specificity, we tested the ability of cognate and non-cognate ligands of CCR5, including CCL2, CCL3, CCL5, CCL7, CCL8, and CCL19, to induce signaling at M1-CCR5, M1-CCR5-1like, and M1-CCR1. As expected, M1-CCR5 and M1-CCR5-1like responded to the same ligands, namely only CCR5 cognate ligands, and M1-CCR1 responded to CCR1 cognate ligands as well as CCL2, as expected from the literature [8] (Figure 9).

### 2.7. Boosting by Basic Peptides Is Independent of Ligand Oligomerization State

**Figure 10:**
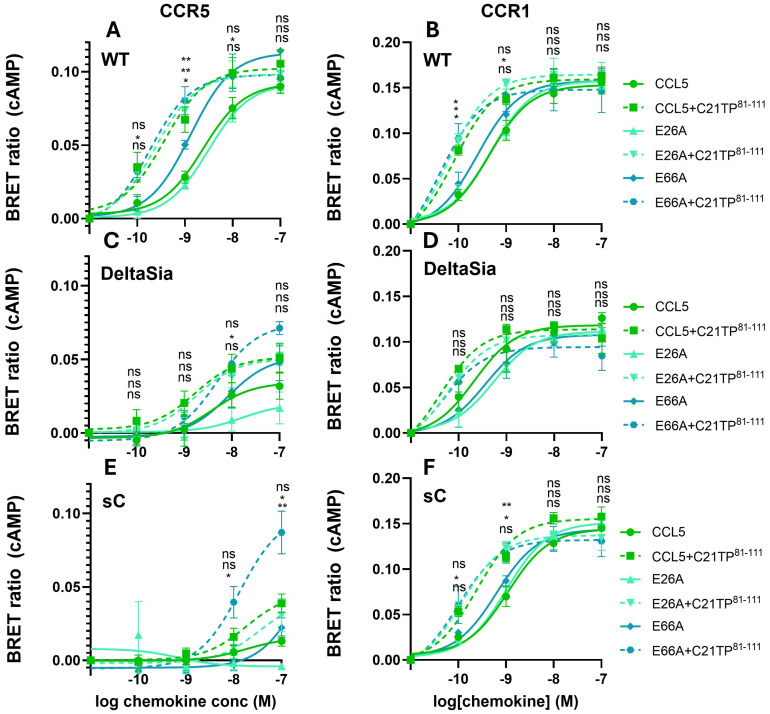
Signaling by CCL5 obligate dimer/tetramer versions is boosted similar to WT CCL5 ligand. Ligand-induced Gα_i_ signaling via CCR5 in HEK293 WT cells transfected as in Figure 1. As can be seen, signaling induced by all three ligands (CCL5, E26A, and E66A) via CCR5 is drastically reduced from WT to DeltaSia to sC cells (**A**,**C**,**E**), whereas all three ligands induce potent signaling responses via CCR1 in all three cell lines (**B**,**D**,**F**). Ligand-induced signaling via both CCR5 and CCR1 is boosted by C21TP^81-111^ in WT cells, a tendency that remains across in DeltaSia and sC cell lines. Interestingly, E66A signaling via CCR5 is significantly boosted by C21TP^81-111^ in sC cells. Statistical significances were determined by two-way ANOVA with Sidak’s multiple comparisons test (n = 3). ** *p* ≤ 0.01, * *p* ≤ 0.05, ns = non-significant.

As boosting of CCL5-induced signaling with C21TP^81-111^ was observed in cells with and without GAGs and in cells expressing site-specific reductions in CCR5 N-terminus O-glycosylation and also in cells lacking overall O-glycosylation, we tested if boosting might rely on disruption of the CCL5 oligomeric structure. Employing mutant versions of CCL5, E26A (obligate tetramer), and E66A (obligate dimer), we were able to show that obligate dimer and tetrameric versions of CCL5 maintain WT-like signaling via CCR5 and CCR1 and that boosting occurs with all three versions of CCL5 in WT HEK293 cells (Figure 10A,B). Signaling for all three versions of CCL5 via CCR1 remains high in DeltaSia and sC cells, with a tendency for boosting with C21TP^81-111^ in DeltaSia cells and significant boosting in sC (Figure 10D,F). Signaling induced by CCL5, E26A, and E66A via CCR5 was very low in both DeltaSia and sC cells, and boosting by C21TP^81-111^ was lost (Figure 10C,E). Interestingly, E66A had a tendency to be more efficient in inducing signaling via CCR5 in both WT and DeltaSia cells. Signaling induced by all three ligands was low in sC cells. On the other hand, boosting of E66A-induced signaling by C21TP^81-111^ was significant at both 10 and 100 nM E66A (Figure 10C,E).

## 3. Discussion

### 3.1. Basic Peptides Boost the Action of Basic but Not Acidic Chemokines

Our study has concentrated on the electrostatic interactions between positively charged chemokines and negatively charged cell and receptor entities and how competing electrostatic interactions affect chemokine receptor engagement. We have focused on the interplay between inherent negative charge (Asp/Glu content) and post-translational modifications of receptor N-termini and negatively charged GAGs/proteoglycans in/on the surrounding cell membrane. We have also investigated the shielding effect of basic boosting peptides and how it aids basic chemokine signaling. CCR1 and CCR5 are both expressed by immature DCs and they share multiple chemokine ligands. We wanted to know if these receptors are equally dependent on PTMs for initial ligand capture and whether signaling induced by the more basic chemokines could be aided by basic peptides. Assessing chemokine-induced signaling via CCR1 and CCR5 in CHO cells, we observed that the more basic the chemokine, the more boosting was exerted by basic peptides. Thus, signaling induced by CCL5 via CCR5 and CCR1 was boosted the most, whereas signaling induced by the acidic chemokine CCL3 through either receptor was unaffected by the presence of basic peptides. Boosting was tested using the basic C-terminal peptide of the chemokine CCL21 (C21TP^81-111^) as well as innate defense peptides in the form of hBD2 and histatin. hBD2 boosted basic chemokine-induced signaling to the same extent as C21TP^81-111^, whereas histatin1, that is less basic, was less potent in its boosting ability.

### 3.2. Signaling via CCR5 Not CCR1 Is Inhibited by GAGs

Earlier studies have shown that C21TP^81-111^ boosting of CCL19 and CCL21 signaling induced at CCR7 occurred independent of cell GAG status. Employing GAG knockout (GAG-minus) and wild-type CHO cells (GAG-plus), we found that, overall, the boosting effect of C21TP^81-111^ was maintained in both cell lines. Nevertheless, whereas CCL5 signaling through CCR5 seems to be drastically boosted in the presence of C21TP^81-111^ in GAG-plus cells the boosting effect was seemingly less in GAG-minus cells, an effect most likely explained by the overall highly increased signaling induced by CCL5 alone in GAG-minus vs. GAG-plus cells. The potency of CCL5 increased significantly in GAG-minus cells (LogEC50: −9627 ± 0.070) compared to GAG-plus cells (LogEC50: −7.522 ± 0.639). In contrast, CCL5 induced signaling with a higher potency at CCR1 than at CCR5 and the potency was less affected by cell GAG status in GAG-minus cells (LogEC50: −9.42 ± 0.131) compared to GAG-plus cells (LogEC50: −8.89 ± 0.133). C21TP^81-111^ boosted CCL5 signaling via CCR1, in both cell lines.

Overall, the CCR5 N-terminus carries less inherent negative charge compared to the CCR1 N-terminus (fewer acidic amino acids). CCR5 has fewer Thr and Ser (potential O-glycosylation sites) compared to CCR1, whereas both receptors have one Asn (potential N-glycosyltion site). As basic chemokines bind to GAGs, there is a possibility that CCR5 is less efficient in competing with GAGs for binding of basic chemokines compared to CCR1.

In GAG-minus cells this could potentially allow high-affinity GAG-binding chemokines to be freed from GAG-bound reservoirs and thereby allow them to interact with CCR5 instead. For CCR1, the very negatively charged N-terminus competes effectively with GAGs for sequestering of positively charged chemokines.

### 3.3. CCR5 Is Highly Dependent on N-Terminal O-Glycosylation for Proper Signaling

We further wanted to assess the contribution of PTMs, that add negative charge to CC receptor N-termini, for efficient chemokine signaling and for the C21TP^81-111^ boosting effect towards basic chemokines. Several demonstrated O-glycosylation sites and tyrosine sulfation sites in the CCR5 N-terminus were evaluated for their importance using N-terminal alanine-substituted receptor versions. Similarly, different HEK cell lines, producing O-glycosylations lacking either the terminal sialic acid residue (HEK-DeltaSia) or lacking O-glycosylations altogether except for the initiating GalNac monosaccharide (HEK-sC), were used to evaluate how O-glycosylation overall (not only in the receptor N-terminus) affects signaling induced by CCL5/8/3 through CCR5 and CCL5/7/3 through CCR1 and the C21TP^81-111^ boosting effect. As expected, compared to CCR5, signaling through CCR1 was less affected by lack of sialic acids and overall O-glycosylation due to its more negatively charged N terminus.

### 3.4. The Initial Contact between a Chemokine and Its Receptor Depends on Non-Selective Electrostatic Interactions Not Definable for Ligand Specificity

According to the two-site, two-step model, a chemokine activates its receptor through a temporal interaction with two distinct sites in the receptor. First, the chemokine core domain (chemokine site 1, CS1) (the N-loop/β-region) binds to the extracellular N-terminal domain of the receptor, also called chemokine recognition site 1 (CRS1), ensuring high-affinity binding. Second, the chemokine N-terminus (CS2) interacts with the 7TM-bundle of the receptor (CRS2), leading to receptor conformational changes and activation [12]. This model has stood the test of time, but more recent studies have revealed that the CS1:CRS1 interaction mainly relies on electrostatic interactions that display allovalency, suggesting that while the interaction is extremely important for initial chemokine capture by the chemokine receptor, it is not a high-affinity interaction and it will allow initial capture of both cognate and non-cognate ligands, with only the cognate ligands proceeding through the high-affinity binding step involving CS2:CSR2, leading to receptor activation [8].

The reliance on electrostatic interactions between basic amino acids in the chemokine and acidic regions in the receptor N-terminus for chemokine receptor binding is illustrated by a study carried out by Hemmerich et al. [27]. To investigate the influence of charge, hydrophobicity, and aromaticity on binding of CCL2 to its cognate receptor CCR2, this group substituted all surface-exposed residues on CCL2 by alanine [27]. Most alanine substitutions, including residues in the CCL2 unstructured N-terminus important for CCL2 signaling via CCR2, did not significantly influence binding. In contrast, a cluster of primarily basic amino acids (R24, K35, K38, K49, and Y13) reduced CCL2 binding to CCR2 by 15–100-fold [27]. Substitution of these basic residues by glutamic acid affected binding tremendously, e.g., while the R24A substitution reduced binding by ~30-fold, R24E reduced binding by ≥1600-fold and the same was observed for K35E [27]. Single substitutions of R18A/E, R19A/E and R29A/E, R30A/E did not have the same dramatic effects. It is possible that the redundance of basic residues in the same area of the chemokine underscores the importance of having a basic residue in these parts of the chemokine. The amino acids found to be important for binding were also needed for subsequent signaling via CCR2 [28], reflecting that binding precedes signaling. Intriguingly, when mutating the N-terminus of CCR2, it was found that substituting acidic residues within the sequence 15-ESGEEVTTFFDYDY-28, D25AD27A, the binding affinity, Kd, changed from 0.066 ± 0.01 nM (mean ± SD) for the WT receptor to ≥3 nM for the D25A/D27A mutant version, an approximately 50-fold reduction in affinity [28]. Thus, overall the initial binding between CCL2 and CCR2 seems to rely heavily on electrostatic interactions between the chemokine and the receptor N-terminus [27]. Interestingly, peptides representing the CCR2 N-terminus, when un-sulfated, did not bind CCL2 in it monomeric nor its dimeric state, whereas sulfated versions of the receptor N-terminus bound both chemokine states, indicating that tyrosine sulfations adding negative charge to CCR2 are of utmost importance for initial chemokine binding to the receptor N-terminus [29]. For another ligand of CCR2, CCL7, tyrosine sulfation of the peptide corresponding to the receptor N-terminus increased chemokine binding by up to 36-fold [30].

CCR5, in contrast to CCR1, has an N-terminus that is sparse in acidic amino acids but has multiple tyrosine sulfation sites as well as O-glycosylation sites, that might increase the negative charge of the receptor N-terminus through post-translational modifications. CCL5 affinity towards peptides corresponding to the CCR5 receptor N-terminus increases by ≥100-fold if the peptides are sulfated on Y10 and Y14 [30], and the same tyrosine sulfation is needed for HIV1env protein binding [31] and viral entry [32].

Another study indicating that the electrostatic interactions formed between the chemokine core domain (CS1) and the chemokine receptor N-terminus (CSR1) are weak and not determining for ligand discrimination came from Sanchez et al. [8]. Their data showed that cognate ligands of CCR1 including CCL5, CCL7, and CCL8 but also ligands that are not considered cognate ligands, e.g., CCL2, bound to a peptide corresponding to the N-terminus of CCR1 (METPNTTEDYDTTTEFDYGDATPSQKVNE) and with higher affinity to peptide versions that were tyrosine sulfated on the underlined residues. CCL2, CCL5, and CCL7 all bound to the fully sulfated version of the CCR1 N-terminal peptide with an affinity of 100 nM, indicating that this interaction does not discriminate between cognate and non-cognate ligands [8]. Strong binding to the entire CCR1 receptor was only observed with cognate ligands, leading Stone et al. to conclude that regions other than the CCR1 N-terminus, most likely the 7TM bundle forming the ligand binding pocket, contribute to ligand specificity.

Based on their data, Sanchez et al. suggested that the two-site, two-step interaction model should be elaborated to a three-step model, in which the first step (a) involves a low-affinity, non-specific, and reversible binding of CS1 in the chemokine to CRS1, followed by (b) a specific rate-limiting, high-affinity interaction between CS2 and CRS2, with (c) docking of the chemokine N-terminus into the receptor binding pocket leading to receptor conformational changes and activation [8].

### 3.5. PTMs Could Add Another Layer in the Control of Chemokine Receptor Activity

Overlapping expression patterns of chemokine receptors with shared ligands could very well represent yet another redundant system in place to secure proper control of immune regulation. However, another explanation could be that receptors that have one or more ligands in common like CCR1 and CCR5, although expressed by the same type of cell, may be under differential control, due to their differential dependence on PTMs, that changes in immune cells going from immature to mature states and dependent on, e.g., the activating substances [33].

CCR1 and CCR5 are both expressed in high amounts on the surface of immature DCs [34]. Upon maturation, DCs, although they still express the mRNA, do not express CCR1 and CCR5 at their surface, but upregulate CXCR4 and CCR7 expression [34]. Naïve T cells express CXCR4 and CCR7, but only activated T cells express CCR1 and CCR5 [35]. Thus, in many cases immune cells at different activation stages express multiple receptors with shared ligands, indicating that other levels of regulations could be important to allow immune cells to differentially respond to ligands for which they express more than one receptor. An example of such intricate regulation explored in depth involves CCR7 expression and how PTM modification of this receptor is regulated by the activating agent [33]. Thus, monocyte-derived DCs (moDCs) upregulate CCR7 upon maturation, and in the same timespan they dramatically upregulate expression of ST8Sia IV, but only in response to immunogenic signals and not, e.g., tolerogenic signals [33]. Thus, 2 days of Toll-like receptor-4 (TLR4) triggering with LPS, moDCs display heavily increased PolySia expression, whereas maturation with PolyI:C (a synthetic dsRNA TLR3 agonist) or R848 (selective TLR7/8 activating ligand) does not trigger nearly the same PolySia expression [33]. This increase in PolySia expression is necessary for CCL21-triggered chemotaxis by DCs [21,33]. If the same type of PTM-controlled differential receptor activation mode is also relevant for receptors like CCR5 needs remains to be seen, but it is probably very likely. The activation of human naïve CD4+ T cells leads to increased de novo sialylation and overexpression of the genes coding for polysialyltransferases ST8Sia II and ST8Sia IV, indicating that CD4+ T cells have the ability to synthesize PolySia [36]. Thus, the differential ability of ligands like CCL5 to activate chemokine receptors expressed by activated T cells could be regulated at the PTM level with PTM changes possibly orchestrated by the type of activation a T cell receives.

### 3.6. Boosting Effect of Basic Peptides Is Not through a Disaggregating Effect on Chemokine Oligomers

In the current study we were unable to expose the underlying mechanism of basic peptide boosting potential towards basic chemokine-induced signaling via CCR1 and CCR5. Thus, boosting remained in GAG-minus cells and for the most basic chemokine, CCL5, also in DeltaSia and Sc Hek293 cells, despite an overall decrease in signaling in both the presence and absence of basic peptides in these cells. CCL5 forms higher-order oligomers [37] with increased GAG affinity compared to disaggregating CCL5 mutant versions (E26A/S and CCL5 E66A/S) [37]. The interface involved in oligomerization includes a hydrophobic interface involving Y27, F28, I62, and L65 as well as electrostatic interactions at the dimer–dimer interface involving K25, E26, E66, and R44 [38]. CCL5 E26 and E66 mutants (substituted with Ala or Ser) display disaggregating effects, creating versions of CCL5 that are dimeric or tetrameric in nature. To see if the boosting of CCL5 could involve disaggregation of oligomeric CCL5, we also tested the effect of C21TP^81-111^ on signaling induced by CCL5 E26A and CCL5 E66A mutants. As shown in Figure 10, CCL5, CCL5 E26A, and CCL5 E66A mutants induce signaling of equal potency and efficacy at CCR5 and boosting by C21TP^81-111^ remains for all three CCL5 variants in WT cells. Highly interestingly, the CCL5 E66A mutant that has been shown by Dyer et al. to have the lowest affinity towards GAGs (heparin and HS) [37] was seemingly the most potent ligand of CCR5 and signaling was boosted in the presence of C21TP^81-111^ in DeltaSia and sC. A possible explanation could be that when CCL5 is in an obligate dimeric form and thus has very low GAG affinity, it has a better chance at binding to the CCR5 N-terminus as the receptor does not have to compete for CCL5 binding to the cell surroundings.

Again, CCL7 that is also boosted by basic peptides is an obligate monomer [39], and boosting by basic peptides involving a mechanism disrupting the formation of higher-order oligomers is unlikely. In general, from the present data, it seems that the boosting mechanism is complex and that it is likely to involve interference with multiple electrostatic interactions between basic chemokines and their receptors as well as the surrounding milieu affecting chemokine docking (N-terminus shielding effect) and availability (cooperation effect).

## 4. Materials and Methods

FBS, penicillin/streptomycin, glutamine, glucose, forskolin, poly-D-lysine hydrobromide, 37% formaldehyde, and mouse monoclonal primary anti-FLAG M1 antibody (Cat. No. F3040) were from Sigma (St. Louis, MO, USA). Human CCL2, CCL3, CCL5, CCL7, CCL8, and CCL19 were from Peprotech (Rocky Hill, NJ, USA). DMEM, PBS, OPTI-MEM trypsin, and Lipofectamine 2000 were from Thermo Scientific (Waltham, MA, USA). Coelenterazine was from Nanoligth (Pinetop, AZ, USA). C21TP^81-111^, histatin, and hBD2 were from Caslo (Lyngby, Denmark). Goat anti-mouse IgG (H + L) antibody (Cat. No. 31430) was from Invitrogen (Waltham, MA, USA). TMB PLUS2 substrate was from Kementec (Taastrup, Denmark). CCL5 E26A and CCL5 E66A were synthesized by Francis Petersen and Brian F. Volkman. Briefly, the E26A and E66A variants of CCL5 were cloned into a pET28a expression vector containing an N-terminal 6xHis-SUMO3 tag and expressed in BL21 (DE3) *E. coli*. Cells were grown at 37 °C in Luria–Bertani media containing 50 μg/mL kanamycin to an OD_600_ of 0.9 before induction with 0.5 mM isopropyl-β-D-thiogalactopyranoside (IPTG). The cultures were grown for an additional 5 h before the cells were pelleted by centrifugation at 5000× *g* and stored at −20 °C.

Bacterial pellets were resuspended in lysis buffer (50 mM Na_2_PO_4_ (pH 8.0), 300 mM NaCl, 10 mM imidazole, 1 mM phenylmethylsulphonyl fluoride, and 0.1% (*v*/*v*) 2-mercaptoethanol (β–ME)). Resuspended cells were lysed via sonication and clarified by centrifugation at 20,000× *g* for 20 min. The lysate supernatant was discarded, and the remaining insoluble pellet was dissolved in Buffer AD (6 M guanidinium chloride, 50 mM Na_2_PO_4_ pH 8.0, 300 mM NaCl, 10 mM imidazole) with 0.1% (*v*/*v*) β–ME. The insoluble pellets were resuspended by sonication and again clarified by centrifugation at 20,000× *g* for 20 min. The resulting supernatant was loaded onto a 5 mL Ni-NTA column equilibrated in Buffer AD using an AKTA-Start system Cytiva (GE Healthcare, Marlborough, MA, USA). The column was washed with Buffer AD, and terminal 6xHis-SUMO3-Chemokine was eluted using Buffer BD (6 M guanidinium, 50 mM sodium acetate pH 4.5, 300 mM NaCl, and 10 mM imidazole). Chemokine was refolded via dropwise dilution into a 6-fold greater volume of Refold Buffer (50 mM Tris pH 8.0) with the addition of 10 mM cysteine and 0.5 mM cystine. Refolded chemokine was concentrated in an Amicon Stirred Cell concentrator (Millipore Sigma) using a 10 kDa membrane. Chemokine was diluted 4× with Refold Buffer and ULP1 added to cleave the N-terminal 6xHis-SUMO3-tag. The AKTA-Start system was used to load the cleaved protein onto a 5 mL column of SP Sepharose Fast Flow resin (GE Healthcare) equilibrated in Refold Buffer. The column was washed with Cation Buffer A (50 mM Tris pH 8.0) and chemokine was eluted using Cation Buffer B (50 mM Tris pH 8.0 containing 2 M NaCl). Chemokines were purified to >99% homogeneity using reverse-phase HPLC.

Cell culturing: The CHO-K1 cell line (Sigma-Aldrich) and a CHO-K1 cell line completely deficient in GAG synthesis due to lack of endogenous B4GalT7 activity [40] (CHO-pgsB 618, referred to as GAG-minus) were grown in RPMI with 10% FBS and penicillin/streptomycin. HEK293 cells were grown in DMEM with 10% FBS, glutamine and penicillin/streptomycin.

We used HEK293 “Wild Type” (WT) (parental line), HEK293 “Simple Cell” (sC) (C1GALT1 KO), and HEK293 “DeltaSia” (DeltaSia) (ST3GAL1/2 KO). All cells were maintained in a humidified incubator at 37 °C with 5% CO_2_. Cells were passaged every 3rd day and the passage number did not exceed 40.

BRET measurements of Gα_i_ signaling and β-arrestin recruitment: Cells were seeded in 6-well plates (500,000 cells/well) and transiently transfected the following day. For BRET measurements of Gα_i_ signaling, cells were transfected with vectors encoding the human chemokine receptors as indicated (160 ng/well) and the CAMYEL sensor (cAMP sensor, YFP-Epac-RLuc) [41] (840 ng/well) using Lipofectamine (6 µL/well). Briefly, DNA was dissolved in 125 µL OPTI-MEM and Lipofectamine was dissolved in 125 µL OPTI-MEM. The DNA and Lipofectamine solutions were combined into a transfection mixture. The transfection mixture was added dropwise to cells incubated in OPTI-MEM (1 mL/well). Transfections were terminated after 5 h by adding 1 mL of fresh culture medium on top of the OPTI-MEM. The following day the cells were loosened from the plates in PBS with 5 mM glucose and subsequently aliquoted in white 96-well iso plates (~25,000 cells/well). When using C21TP variants and defense peptides (hBD2 and histatin), these were added to a final concentration of 10 μM/well, while an equal amount of PBS was added to control cells. The bioluminescence substrate coelenterazine was added to a final conc. of 5 µM. After 10 min, varying ligand concentrations were added. For BRET measurements of Gα_i_ signaling, forskolin was added 5 min after the addition of the ligand, to a final conc. of 5 µM. The plates were kept in the dark at all times. A Perkin Elmer Envision machine was used for measuring the emission signals at 530 and 480 nm. The BRET signal is determined as the ratio eYFP (530 nm)/Rluc (480 nm).

BRET principle with CAMYEL: For quantification of signaling initiated immediately downstream of receptor activation, we used the cAMP binding CAMYEL sensor to perform bioluminescence resonance energy transfer (BRET) based assays. In this assay we measure changes in intracellular cAMP levels in response to receptor activation. Activation of chemokine receptors lead to a decrease in cAMP through inhibition of adenylate cyclase (AC). In the current setup we initially add forskolin that stimulates AC directly to induce an increase in intracellular cAMP, and we then measure the ability of a ligand (chemokine) to counteract the forskolin-induced increase in cAMP. The cAMP sensor (CAMYEL cAMP sensor, YFP-Epac-RLuc) carries the donor *Renilla luciferase* (Rluc) and the acceptor YFP. Coelenterazine is a substrate for Rluc that will emit light at around 475 nm upon enzymatic cleavage catalyzed by Rluc. When Rluc and YFP are in close proximity, the energy from the emitted light from coelenterazine can be transferred to YFP, resulting in an excitation of YFP, leading to a BRET signal around 525 nm. EPAC is a cAMP binding protein that changes conformation upon cAMP binding. During low cAMP concentrations, Rluc and YFP are near each other, and a strong BRET signal at 525 nm is obtained. When cAMP levels increase, cAMP will bind to EPAC, creating a conformational change, separating Rluc and YFP, reducing the BRET ratio, and increasing the emission from Rluc at 475 nm. Thus, by measuring the ratio between the cAMP-bound (475 nm) and the cAMP-free (525 nm) state of CAMYEL we obtain a measure of receptor activation. The BRET ratio is determined as the ratio between YFP/Rluc, and an increase in BRET ratio indicates a decrease in cAMP and thus increased chemokine receptor activation.

ELISA: The cell surface expression level of M1-CCR1, M1-CCR5, and M1-CCR5-1like was estimated by cell-based ELISA. Briefly, 20 000 cells (WT, DeltaSia, sC) were seeded per well in a 96-well plate coated with poly-D-lysine. The next day the cells were transfected with a total of 25, 10, 2.5, and 0 ng receptor construct per well (duplicates of each construct for all three cell lines). On day 3, the cells were washed once in PBS and fixed in 3.7% formaldehyde in PBS for 10 min at RT. The plate was washed three times in PBS and the wells were blocked in PBS with 2% *w*/*v* BSA (PBS/BSA) for 30 min. Primary anti:M1 antibody in PBS/BSA was added and left for 2 h on a shaking table (rpm). The plate was washed twice in PBS, secondary goat anti-mouse HRP-conjugated antibody was added in PBS/BSA, and it was left for 2 h on a shaking table. The plate was washed twice in PBS and 150 microliters of TMB substrate was added per well. The reaction was stopped by adding 100 µL per well of 0.2 M H_2_SO_4_. The plate absorbance was measured using an Envision plate reader at 450 nm wavelength.

Fluorescence polarization (FP) assay: The carboxyfluorescein (FAM)-labeled peptides representing the CCR1 and CCR5 N-termini functioned as the probes for quantifying the interaction between the receptor N-terminus and C21TP^81-111^. The probe was diluted in FP buffer (1:3, PBS:MilliQ) and 10 µL was added to a final concentration of 250 nM per well in a black 384-well plate (Corning). The C21TP^81-111^ peptide (2 mM) was diluted using a two-fold dilution series; 30 µL of the peptide was added to each well to final concentrations ranging from 12.21 nM to 100 µM. Blanks containing 10 µL probe and 30 µL FP buffer were included. The plate was spun down at 1000 rpm for 1 min and the FP signal was measured on a Flex station using SoftMax Pro software 7.0. Each experiment was conducted in triplicate. The binding curves were generated by plotting the FP signal, expressed as millipolarization units (mP), against C21TP^81-111^ concentration. The dissociation constant (*K*_d_) value was calculated using GraphPad Prism 9. 

Probes:

CCR1: (5,6-FAM)-QEDYDTTTEFDYGDATPCQKVNER,
CCR5: (5,6-FAM)-ADYQVSSPIYDINYYTSEP.

Mutagenesis: Mutations were introduced into the CCR5 WT pcDNA 3.1^+^ expression vector by PCR using the QuikChange™ site-directed mutagenesis kit (Stratagene, La Jolla, CA, USA) according to the manufacturer’s instructions. All mutations were verified by DNA sequence analysis.

### 4.1. Primers

S/T to A substitutions:

CCR5-S6A-FP: ATG GAT TAT CAA GTG GCA AGT CCA ATC TAT GAC;
CCR5-S6A-RP: GTC ATA GAT TGG ACT TGC CAC TTG ATA ATC CAT;
CCR5-S7A-FP: ATG GAT TAT CAA GTG TCA GCT CCA ATC TAT GAC;
CCR5-S7A-RP: GTC ATA GAT TGG AGC TGA CAC TTG ATA ATC CAT;
CCR5-S6S7AA-FP: ATG GAT TAT CAA GTG GCA GCT CCA ATC TAT GAC;
CCR5-S6S7AA-RP: GTC ATA GAT TGG AGC TGC CAC TTG ATA ATC CAT;
CCR5-T16S17AA-FP: GAC ATC AAT TAT TAT GCA GCG GAG CCC TGC CAA;
CCR5-T16S17AA-RP: TTG GCA GGG CTC CGC TGC ATA ATA ATT GAT GTC.

Y to A substitutions:

CCR5-Y10A-FP: GTG TCA AGT CCA ATC GCT GAC ATC AAT TAT TAT ACA TCG;
CCR5-Y10A-RP: CGA TGT ATA ATA ATT GAT GTC AGC GAT TGG ACT TGA CAC;
CCR5-Y14Y15AA-FP: GCA GGG CTC CGA TGT AGC AGC ATT GAT GTC ATA GAT;
CCR5-Y14Y15AA-RP: ATC TAT GAC ATC AAT GCT GCT ACA TCG GAG CCC TGC.

### 4.2. N-Terminus Swap-In

CCR5-1-like FP: TAT CAA GTG TCA AGT GAG GAC TAT GAC ACG ACC ACA GAGTTT GAC CCA ATC TAT GAC ATC.

CCR5-1-like RP: GAT GTC ATA GAT TGG GTC AAA CTC TGT GGT CGT GTC ATA GTC CTC ACT TGA CAC TTG ATA.

## 5. Conclusions

In the current study we reveal that CCR5 depends on N-terminal O-glycosylation for proper signaling, whereas CCR1 signals independent of this type of PTM in response to cognate ligands. We hypothesize that the less acidic CCR5 N-terminus is unable to compete with surrounding GAGs for chemokine capture.

Basic peptides seem to boost signaling induced at CCR1 and CCR5 by their shared basic chemokine CCL5 as well as cognate basic ligands CCL8 and CCL7, respectively, whereas no boosting of the signaling induced by the acidic chemokine CCL3 is observed with either receptor. Boosting seems to rely on a mechanism that is independent of GAGs, but we cannot rule out that the peptides also boost signaling via a mechanism related to the chemokine cooperation model, where abundant basic chemokines may free less abundant basic chemokines from a GAG-bound reservoir, leading to a higher local concentration of ligand available for receptor engagement.

As the basic peptides have been shown to have affinity for the naked (unmodified) CCR1 receptor N-terminus, but not for the naked CCR5 receptor N-terminus, it makes sense that boosting is lost for ligand-induced signaling at CCR5 in DeltaSia and sC cells, whereas boosting is preserved in these cells for ligand-induced signaling at CCR1.

**Figure 11:**
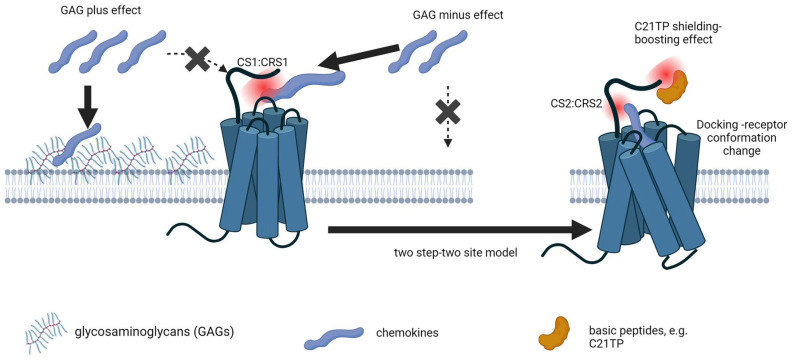
Schematic representation of the two-step, two-site model of chemokine–receptor interaction with GAG and basic peptide modulation. Due to their negatively charged nature, GAGs on the cell surface and in ECM sequester basic chemokines, creating localized chemokine stores (arrow 1) and less chemokine is captured by the CRS1 (stippled line 1 crossed out). In contrast to chemokine receptors with acidic N-termini, e.g., CCR1, receptors with less acidic N-termini, such as CCR5, rely on post-translational modifications (PTMs) to increase their net negative charge for effective ligand capture (CS1:CRS1 interaction). In the absence of GAGs, native CCR5 signals potently, probably because the surroundings are less acidic, allowing for CCR5 to compete better for ligand capture (arrow 2), as chemokines do not bind strongly to GAG-free cell surface (stippled line 2 crossed out). As published previously, basic peptides, like the C-terminal peptide of CCL21 (C21TP), binds in a dose-dependent manner to acidic receptor N-termini (e.g., CCR7). In the current study we have shown that basic peptides, including C21TP, bind to a peptide corresponding to the unmodified native CCR1 N-terminus but not a peptide corresponding to the unmodified native CCR5 N-terminus. Basic peptides seem to aid the transition from CS1 interaction to CS2 insertion by shielding the acidic receptor N-terminus, allowing for an easier transition from CS1:CRS1 to CS2: CRS2 interaction mode.

The proposed effects of GAGs and basic peptides have been outlined in Figure 11.

## Data Availability

The datasets generated during and/or analyzed during the current study are available from the corresponding author on reasonable request.

## Supplementary Materials

The following supporting information can be downloaded at: https://www.mdpi.com/article/10.3390/ijms251910854/s1.

## References

[1] Bachelerie F., Ben-Baruch A., Burkhardt A.M., Combadiere C., Farber J.M., Graham G.J., Horuk R., Sparre-Ulrich A.H., Locati M., Luster A.D. (2013). International Union of Basic and Clinical Pharmacology. [corrected]. LXXXIX. Update on the extended family of chemokine receptors and introducing a new nomenclature for atypical chemokine receptors. Pharmacol. Rev..

[2] Steen A., Larsen O., Thiele S., Rosenkilde M.M. (2014). Biased and g protein-independent signaling of chemokine receptors. Front. Immunol..

[3] Corbisier J., Gales C., Huszagh A., Parmentier M., Springael J.Y. (2015). Biased signaling at chemokine receptors. J. Biol. Chem..

[4] Metzemaekers M., Van D.J., Mortier A., Proost P. (2016). Regulation of Chemokine Activity—A Focus on the Role of Dipeptidyl Peptidase IV/CD26. Front. Immunol..

[5] Leach K., Charlton S.J., Strange P.G. (2007). Analysis of second messenger pathways stimulated by different chemokines acting at the chemokine receptor CCR5. Biochem. Pharmacol..

[6] Verhallen L., Lackman J.J., Wendt R., Gustavsson M., Yang Z., Narimatsu Y., Sørensen D.M., Lafferty K.M., Gouwy M., Marques P.E. (2023). “Glyco-sulfo barcodes” regulate chemokine receptor function. Cell Mol. Life.

[7] Sanchez J., Lane J.R., Canals M., Stone M.J. (2019). Influence of Chemokine N-Terminal Modification on Biased Agonism at the Chemokine Receptor CCR1. Int. J. Mol. Sci..

[8] Sanchez J., Huma Z.E., Lane J.R., Liu X., Bridgford J.L., Payne R.J., Canals M., Stone M.J. (2019). Evaluation and extension of the two-site, two-step model for binding and activation of the chemokine receptor CCR1. J. Biol. Chem..

[9] Verkaar F., Van Offenbeek J., Van Der Lee M.M.C., Van Lith L.H.C.J., Watts A.O., Rops A.L.W.M.M., Aguilar D.C., Ziarek J.J., Van Der Vlag J., Handel T.M. (2014). Chemokine cooperativity is caused by competitive glycosaminoglycan binding. J. Immunol..

[10] de Paz J.L., Moseman E.A., Noti C., Polito L., von Andrian U.H., Seeberger P.H. (2007). Profiling heparin-chemokine interactions using synthetic tools. ACS Chem. Biol..

[11] Patel D.D., Koopmann W., Imai T., Whichard L.P., Yoshie O., Krangel M.S. (2001). Chemokines have diverse abilities to form solid phase gradients. Clin. Immunol..

[12] Crump M.P., Gong J.H., Loetscher P., Rajarathnam K., Amara A., Arenzana-Seisdedos F., Virelizier J.L., Baggiolini M., Sykes B.D., Clark-Lewis I. (1997). Solution structure and basis for functional activity of stromal cell-derived factor-1; dissociation of CXCR4 activation from binding and inhibition of HIV-1. EMBO.

[13] Hancock R.E., Haney E.F., Gill E.E. (2016). The immunology of host defence peptides: Beyond antimicrobial activity. Nat. Rev. Immunol..

[14] Liu A.Y., Destoumieux D., Wong A.V., Park C.H., Valore E.V., Liu L., Ganz T. (2002). Human beta-defensin-2 production in keratinocytes is regulated by interleukin-1, bacteria, and the state of differentiation. J. Investig. Dermatol..

[15] Schumann K., Lämmermann T., Bruckner M., Legler D.F., Polleux J., Spatz J.P., Schuler G., Förster R., Lutz M.B., Sorokin L. (2010). Immobilized chemokine fields and soluble chemokine gradients cooperatively shape migration patterns of dendritic cells. Immunity.

[16] Lorenz N., Loef E.J., Kelch I.D., Verdon D.J., Black M.M., Middleditch M.J., Greenwood D.R., Graham E.S., Brooks A.E., Dunbar P.R. (2016). Plasmin and regulators of plasmin activity control the migratory capacity and adhesion of human T cells and dendritic cells by regulating cleavage of the chemokine CCL21. Immunol. Cell Biol..

[17] Loef E.J., Sheppard H.M., Birch N.P., Dunbar P.R. (2022). Plasminogen and plasmin can bind to human T cells and generate truncated CCL21 that increases dendritic cell chemotactic responses. J. Biol. Chem..

[18] Hauser M.A., Kindinger I., Laufer J.M., Späte A.-K., Bucher D., Vanes S.L., Krueger W.A., Wittmann V., Legler D.F. (2016). Distinct CCR7 glycosylation pattern shapes receptor signaling and endocytosis to modulate chemotactic responses. J. Leukoc. Biol..

[19] Jørgensen A.S., Brandum E.P., Mikkelsen J.M., Orfin K.A., Boilesen D.R., Egerod K.L., Moussouras N.A., Vilhardt F., Kalinski P., Basse P. (2021). The C-terminal peptide of CCL21 drastically augments CCL21 activity through the dendritic cell lymph node homing receptor CCR7 by interaction with the receptor N-terminus. Cell Mol. Life Sci..

[20] Brandum E.P., Jørgensen A.S., Calvo M.B., Spiess K., Peterson F.C., Yang Z., Volkman B.F., Veldkamp C.T., Rosenkilde M.M., Goth C.K. (2022). Selective Boosting of CCR7-Acting Chemokines; Short Peptides Boost Chemokines with Short Basic Tails, Longer Peptides Boost Chemokines with Long Basic Tails. Int. J. Mol. Sci..

[21] Kiermaier E., Moussion C., Veldkamp C.T., Gerardy-Schahn R., De Vries I., Williams L.G., Chaffee G.R., Phillips A.J., Freiberger F., Imre R. (2016). Polysialylation controls dendritic cell trafficking by regulating chemokine recognition. Science.

[22] Rey-Gallardo A., Delgado-Martin C., Gerardy-Schahn R., Rodriguez-Fernandez J.L., Vega M.A. (2011). Polysialic acid is required for neuropilin-2a/b-mediated control of CCL21-driven chemotaxis of mature dendritic cells and for their migration in vivo. Glycobiology.

[23] Rey-Gallardo A., Escribano C., Delgado-Martin C., Rodriguez-Fernandez J.L., Gerardy-Schahn R., Rutishauser U., Corbi A.L., Vega M.A. (2010). Polysialylated neuropilin-2 enhances human dendritic cell migration through the basic C-terminal region of CCL21. Glycobiology.

[24] Goth C.K., Petaja-Repo U.E., Rosenkilde M.M. (2020). G Protein-Coupled Receptors in the Sweet Spot: Glycosylation and other Post-translational Modifications. ACS Pharmacol. Transl. Sci..

[25] Bannert N., Craig S., Farzan M., Sogah D., Santo N.V., Choe H., Sodroski J. (2001). Sialylated O-glycans and sulfated tyrosines in the NH2-terminal domain of CC chemokine receptor 5 contribute to high affinity binding of chemokines. J. Exp. Med..

[26] Kessler N., Akabayov S.R., Moseri A., Cohen L.S., Sakhapov D., Bolton D., Fridman B., Kay L.E., Naider F., Anglister J. (2020). Allovalency observed by transferred NOE: Interactions of sulfated tyrosine residues in the N-terminal segment of CCR5 with the CCL5 chemokine. FEBS J..

[27] Hemmerich S., Paavola C., Bloom A., Bhakta S., Freedman R., Grunberger D., Krstenansky J., Lee S., McCarley D., Mulkins M. (1999). Identification of residues in the monocyte chemotactic protein-1 that contact the MCP-1 receptor, CCR2. Biochemistry.

[28] Jarnagin K., Grunberger D., Mulkins M., Wong B., Hemmerich S., Paavola C., Bloom A., Bhakta S., Diehl F., Freedman R. (1999). Identification of surface residues of the monocyte chemotactic protein 1 that affect signaling through the receptor CCR2. Biochemistry.

[29] Tan J.H.Y., Ludeman J.P., Wedderburn J., Canals M., Hall P., Butler S.J., Taleski D., Christopoulos A., Hickey M.J., Payne R.J. (2013). Tyrosine sulfation of chemokine receptor CCR2 enhances interactions with both monomeric and dimeric forms of the chemokine monocyte chemoattractant protein-1 (MCP-1). J. Biol. Chem..

[30] Ludeman J.P., Stone M.J. (2014). The structural role of receptor tyrosine sulfation in chemokine recognition. Brit. J. Pharmacol..

[31] Blanpain C., Doranz B.J., Vakili J., Rucker J., Govaerts C., Baik S.S.W., Lorthioir O., Migeotte I., Libert F., Baleux F. (1999). Multiple charged and aromatic residues in CCR5 amino-terminal domain are involved in high affinity binding of both chemokines and HIV-1 Env protein. J. Biol. Chem..

[32] Farzan M., Chung S., Li W., Vasilieva N., Wright P.L., Schnitzler C.E., Marchione R.J., Gerard C., Gerard N.P., Sodroski J. (2002). Tyrosine-sulfated peptides functionally reconstitute a CCR5 variant lacking a critical amino-terminal region. J. Biol. Chem..

[33] Bax M., van Vliet S.J., Litjens M., Garcia-Vallejo J.J., van Kooyk Y. (2009). Interaction of polysialic acid with CCL21 regulates the migratory capacity of human dendritic cells. PLoS ONE.

[34] Sallusto F., Schaerli P., Loetscher P., Schaniel C., Lenig D., Mackay C.R., Qin S., Lanzavecchia A. (1998). Rapid and coordinated switch in chemokine receptor expression during dendritic cell maturation. Eur. J. Immunol..

[35] Sallusto F., Kremmer E., Palermo B., Hoy A., Ponath P., Qin S., Förster R., Lipp M., Lanzavecchia A. (1999). Switch in chemokine receptor expression upon TCR stimulation reveals novel homing potential for recently activated T cells. Eur. J. Immunol..

[36] Villanueva-Cabello T.M., Gutierrez-Valenzuela L.D., Lopez-Guerrero D.V., Cruz-Munoz M.E., Mora-Montes H.M., Martinez-Duncker I. (2019). Polysialic acid is expressed in human naive CD4+ T cells and is involved in modulating activation. Glycobiology.

[37] Dyer D.P., Salanga C.L., Volkman B.F., Kawamura T., Handel T.M. (2015). The dependence of chemokine-glycosaminoglycan interactions on chemokine oligomerization. Glycobiology.

[38] Wang X., Watson C., Sharp J.S., Handel T.M., Prestegard J.H. (2011). Oligomeric structure of the chemokine CCL5/RANTES from NMR, MS, and SAXS data. Structure.

[39] Wang X., Sharp J.S., Handel T.M., Prestegard J.H. (2013). Chemokine oligomerization in cell signaling and migration. Prog. Mol. Biol. Transl. Sci..

[40] Esko J.D., Weinke J.L., Taylor W.H., Ekborg G., Roden L., Anantharamaiah G., Gawish A. (1987). Inhibition of chondroitin and heparan sulfate biosynthesis in Chinese hamster ovary cell mutants defective in galactosyltransferase I. J. Biol. Chem..

[41] Jiang L.I., Collins J., Davis R., Lin K.M., DeCamp D., Roach T., Hsueh R., Rebres R.A., Ross E.M., Taussig R. (2007). Use of a cAMP BRET sensor to characterize a novel regulation of cAMP by the sphingosine 1-phosphate/G13 pathway. J. Biol. Chem..

