# Supplementary material for: Chemokine Receptor N-Terminus Charge Dictates Reliance on Post-Translational Modifications for Effective Ligand Capture and Following Boosting by Defense Peptides
Source: Int J Mol Sci. 2024 Oct 9;25(19):10854. doi: 10.3390/ijms251910854 (PMC11477141; doi:10.3390/ijms251910854)
Supplement: Supplementary file 1 [file ijms-25-10854-s001.zip › ijms-3219820-supplementary.pdf]

Supplementary Materials:

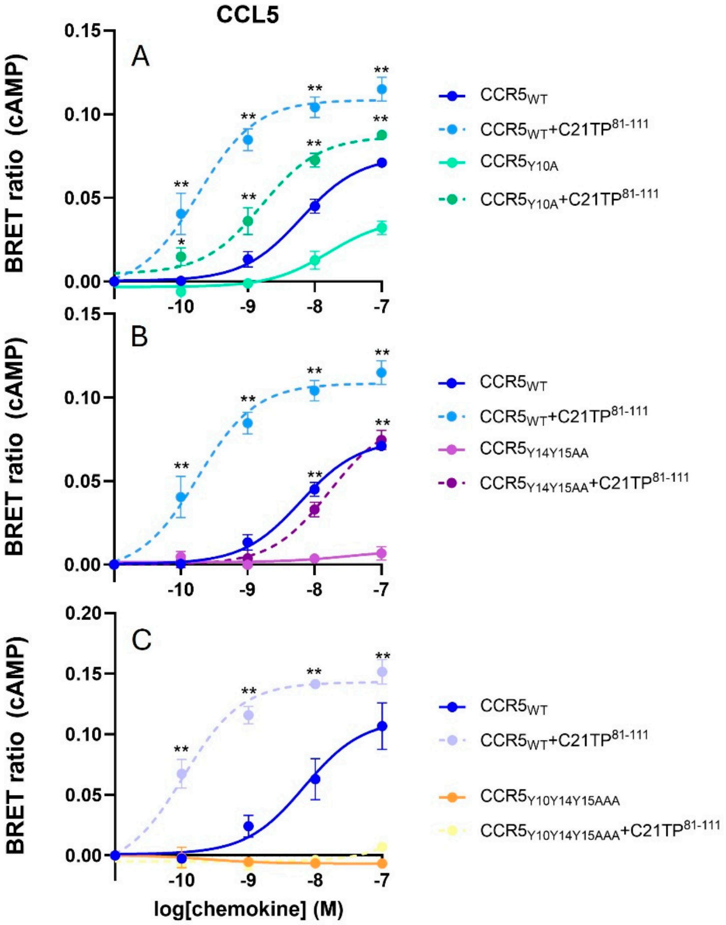

Supplementary Figure S1. Ligand induced $G\alpha_i$-signaling via CCR5 and alanine substituted receptor versions in the absence or presence of $C21TP^{81-111}$ in CHO cells transfected as in figure 1. As can be seen S.figure 1A and 1B, substitution of tyrosine 14 and 15 in CCR5, $CCR5_{Y14Y15AA}$, abolishes signaling induced by CCL5, whereas substitution of tyrosine 10 in CCR5, $CCR5_{Y10A}$,. although it drastically reduces signaling still allows some activity of CCL5. In both cases CCL5 signaling is boosted in the presence of $C21TP^{81-111}$. Substitution of tyrosine 10, 14 and 15 in CCR5, $CCR5_{Y10Y14Y15AAA}$, abolishes signaling induced by CCL5 and boosting by $C21TP^{81-111}$ is no longer possible (S.Fig.1C). Statistical significances were determined by two-way ANOVA with Sidak's multiple comparisons test (n=3). $^{**}P\leq0.01$, $^{*}P\leq0.05$.

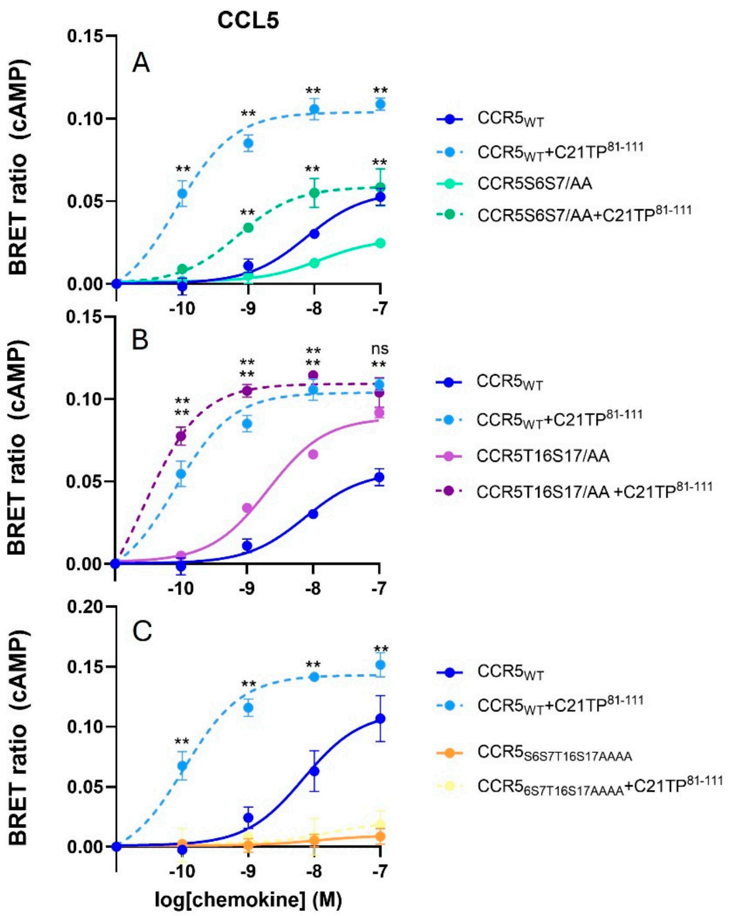

Supplementary Figure S2. Ligand induced Gα_i-signaling via CCR5 and alanine substituted receptor versions in the absence or presence of C21TP$^{81-111}$ in CHO cells transfected as in figure 1. As can be seen in S.figure 2A and 2B, substitution of serine 6 and 7 in CCR5, CCR5$_{S6S7AA}$, reduces signaling induced by CCL5, whereas substitution of threonine 16 and serine 17 in CCR5, CCR5$_{T16S16AA}$, creates a version of CCR5 that is activated more by CCL5 with increases in both ligand potency and efficacy. For all receptor version boosting by C21TP$^{81-111}$ remained. Substitution of serine 6 and 7 and 17 and threonine 16 in CCR5 creates a receptor version, CCR5$_{S6S7T16S16AAAA}$, that has lost its responsiveness to CCL5 in both the presence and absence of C21TP$^{81-111}$. Statistical significances were determined by two-way ANOVA with Sidak's multiple comparisons test (n=3). **P≤0.01, *P≤0.05.
